# Mitogen-Activated Protein Kinases and Reactive Oxygen Species: How Can ROS Activate MAPK Pathways?

**DOI:** 10.1155/2011/792639

**Published:** 2011-02-06

**Authors:** Yong Son, Yong-Kwan Cheong, Nam-Ho Kim, Hun-Taeg Chung, Dae Gill Kang, Hyun-Ock Pae

**Affiliations:** ^1^Department of Anesthesiology and Pain Medicine, Wonkwang University School of Medicine, Iksan 570-749, Republic of Korea; ^2^Department of Cardiovascular Medicine, Wonkwang University Hospital, Iksan 570-711, Republic of Korea; ^3^Department of Biological Science, University of Ulsan, Ulsan 680-749, Republic of Korea; ^4^Professional Graduate School of Oriental Medicine and Hanbang Body-Fluid Research Center, Wonkwang University, Iksan 570-749, Republic of Korea; ^5^Department of Microbiology and Immunology, Wonkwang University School of Medicine, 344-2 Shinyong-dong, Iksan, Chonbuk 570-749, Republic of Korea

## Abstract

Mitogen-activated protein kinases (MAPKs) are serine-threonine protein kinases that play the major role in signal transduction from the cell surface to the nucleus. MAPKs, which consist of growth factor-regulated extracellular signal-related kinases (ERKs), and the stress-activated MAPKs, c-jun NH_2_-terminal kinases (JNKs) and p38 MAPKs, are part of a three-kinase signaling module composed of the MAPK, an MAPK kinase (MAP2K) and an MAPK kinase (MAP3K). MAP3Ks phosphorylate MAP2Ks, which in turn activate MAPKs. MAPK phosphatases (MKPs), which recognize the TXY amino acid motif present in MAPKs, dephosphorylate and deactivate MAPKs. MAPK pathways are known to be influenced not only by receptor ligand interactions, but also by different stressors placed on the cell. One type of stress that induces potential activation of MAPK pathways is the oxidative stress caused by reactive oxygen species (ROS). Generally, increased ROS production in a cell leads to the activation of ERKs, JNKs, or p38 MAPKs, but the mechanisms by which ROS can activate these kinases are unclear. Oxidative modifications of MAPK signaling proteins and inactivation and/or degradation of MKPs may provide the plausible mechanisms for activation of MAPK pathways by ROS, which will be reviewed in this paper.

## 1. Introduction

Mitogen-activated protein kinases (MAPKs) compose a family of protein kinases that play an essential role in relaying extracellular signals from the cell membrane to the nucleus *via* a cascade of phosphorylation events and are negatively regulated by MAPK phosphatases (MKPs) [1]. Diverse cellular functions, ranging from cell survival to cell death, are regulated by MAPK signaling [2]. A number of extracellular and intracellular stimuli have been shown to activate MAPK pathways at cellular levels [3], implying that there may be tight and specific regulation of MAPK activation by a certain stimulus. Interestingly, reactive oxygen species (ROS) can activate MAPK pathways [4], but the mechanism(s) for this effect is unclear. 

Besides MAPKs, other signaling molecules (e.g., protein tyrosine phosphatases, protein tyrosine kinases, and transcriptional factors) can also be activated by ROS [5], suggesting that ROS may have meaningful roles as regulators of cell function or as signaling molecules. Indeed, mounting evidence supports a physiological role for ROS as a “second messenger” in intracellular signaling cascades that control cell growth, proliferation, migration, and apoptosis [5]. 

Because the MAPK pathways mediate both mitogen- and stress-activated signals, there has been significant interest in the regulation of these pathways by ROS. This paper will focus on the putative mechanisms by which ROS can activate MAPK pathways in a cell. 

## 2. ROS

ROS include superoxide anion radical (·O_2_^−^), hydroxyl radicals (·OH), and hydrogen peroxide (H_2_O_2_). H_2_O_2_ is not a free radical and a weaker oxidizing agent than the free radical ·O_2_^−^. However, in the presence of transition metals such as iron or copper, H_2_O_2_ can be oxidized into the extremely reactive and toxic ·OH *via* well-known Fenton reaction. In the cellular systems, ROS are normally counteracted by ubiquitously expressed antioxidant proteins, such as superoxide dismutase (SOD), catalase, glutathione (GSH) peroxidase, thioredoxin, glutaredoxin, and GSH. For example, SOD can convert ·O_2_^−^ into H_2_O_2_, whereas catalase and GSH peroxidase can reduce H_2_O_2_. 

ROS are constantly produced by a number of normal cellular events, with a major source being aerobic respiration, but ROS produced during these events are generally counteracted by several antioxidant proteins [6, 7]. A large amount of ROS can also be produced by inflammatory processes, ionizing radiation, and many chemotherapeutic drugs, and this, if the production of ROS exceeds the capacity of the antioxidant proteins, may cause the so-called “oxidative stress”; in a biological sense, the oxidative stress may be broadly defined as an imbalance between oxidant production and the antioxidant capacity of the cell to prevent oxidative injury [5, 7]. 

Oxidative stress is known to be implicated in many human diseases, including atherosclerosis, cancer, neurodegenerative diseases, and aging [7]. However, there is still a debate whether oxidative stress is a cause or a result of these diseases, largely due to a lack of our understanding of the mechanisms by which ROS function in both normal physiological and disease states. 

ROS are not only injurious to cell survival but also essential to cell signaling and regulation, and this may be dependent on the levels of produced ROS. At high levels, ROS can lead to impaired physiological function through cellular damage of DNA, proteins, phospholipids, and other macromolecules, which can lead to certain human pathologies [8]. At low levels, ROS can alter intracellular redox state, leading to activation of redox-sensitive proteins, and also modify redox-sensitive parts of proteins, potentially inhibiting or increasing their enzymatic activity [9, 10]. H_2_O_2_, with a relatively long half-life, good membrane permeability, and higher intracellular concentration, has been proposed to function as a second messenger [9, 10]. In this regard, it is most likely that H_2_O_2_ may mimic many actions of ROS in a cellular system. 

## 3. MAPKs

**Figure 1:**
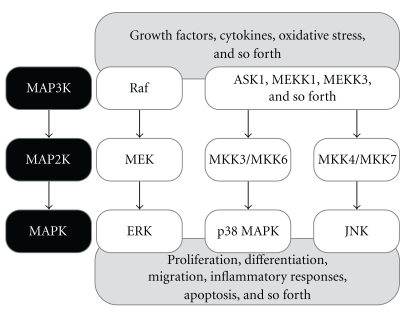
MAPK cascades. MAPK signaling pathways mediate intracellular signaling initiated by extracellular or intracellular stimuli. MAP3Ks phosphorylate MAP2Ks, which in turn phosphorylate MAPKs. Activated MAPKs phosphorylate various substrate proteins (e.g., transcription factors), resulting in regulation of various cellular activities (e.g., proliferation, differentiation, inflammatory responses, and apoptosis). Activation by MAPK signaling cascades is achieved either through a series of binary interactions among the kinase components or through formation of a multiple kinase complex.

The MAPKs comprise a family of ubiquitous proline-directed, protein-serine/threonine kinases, which play an essential role in sequential transduction of biological signals from the cell membrane to the nucleus [11]. In mammalian cells, there are three well-defined subgroups of MAPKs: the extracellular signal regulated kinases (ERKs, including ERK-1 and ERK-2 isoforms), the c-Jun *N*-terminal kinases (JNKs, including JNK-1, JNK-2, and JNK-3 isoforms), and the p38 MAPKs (including p38-*α*, p38-*β*, p38-*γ*, and p38-*δ* isoforms). Each subgroup of MAPKs is activated through a cascade of sequential phosphorylation events, beginning with the activation of MAPK kinase kinases (MAP3Ks). The MAP3Ks phosphorylate and activate a downstream dual-specificity MAPK kinases (MAP2Ks), which in turn stimulate MAPK activity through dual phosphorylation on threonine and tyrosine residues within a conserved tripeptide motif [1, 11]. The well-defined regulation of MAPK signaling pathways is summarized in Figure 1. It should be noted that the three subgroups of MAPKs (i.e., ERKs, JNKs, and p38 MAPKs) are involved in both cell growth and cell death, and the tight regulation of these pathways is paramount in determining cell fate [12]. The deleterious consequences of sustained activation of MAPK pathways may include excessive production of MAPK-regulated genes, uncontrolled proliferation, and unscheduled cell death. 

### 3.1. ERKs

ERK pathway is activated by MAP/ERK Kinase (MEK), which is activated by Raf. Raf, an MAP3K, is activated by the Ras-GTPase, whose activation is induced by receptor tyrosine kinases (RTKs) such as the epidermal growth factor (EGF) receptor [13]. Growth factor receptors are most commonly activated by ligand-induced dimerization or oligomerization that phosphorylates RTKs [14]. Ligand-independent clustering and activation of growth factor receptors in response to ROS have also been well demonstrated [15]. Meves et al. [16] demonstrated that oxidative stress induces EGF receptor activation through RTK phosphorylation and proposed that H_2_O_2_ is a critical mediator required for ligand-independent phosphorylation of growth factor receptors in response to oxidative stress. 

### 3.2. p38 MAPKs

The p38 MAPKs are usually activated in response to inflammatory cytokines, as well as by many other stimuli, including hormones, ligands for G protein-coupled receptors, and stresses such as heat shock and osmotic shock [17]. Two MEK family members, MEK3 (or MKK3) and MEK6 (or MKK6), are highly specific for p38 MAPKs [17]. MKK6 can phosphorylate the four p38 MAPK family members, while MKK3 phosphorylates p38*α*, p38*γ*, and p38*δ*, but not p38*β*. Both will also phosphorylate JNK isoforms [17]. Several MAP3Ks have been shown to trigger p38 MAPK activation, and they include ASK1 (apoptosis signal-regulating kinase 1), DLK1 (dual-leucine-zipper-bearing kinase 1), TAK1 (transforming growth factor *β*-activated kinase 1), TAO (thousand-and-one amino acid) 1 and 2, TPL2 (tumor progression loci 2), MLK3 (mixed-lineage kinase 3), MEKK3 (MEK kinase 3) and MEKK4, and ZAK1 (leucine zipper and sterile-*α* motif kinase 1) [17]. The diversity of MAP3Ks and their regulatory mechanisms may provide the ability to respond to a wide range of stimuli and to integrate p38 MAPK activation with other signaling pathways. It should be noted that some MAP3Ks that trigger p38 MAPK activation can also activate the JNK pathway. 

### 3.3. JNKs

The JNK pathway is known to be activated by cytokines, ligation of a variety of receptors, agents that interfere with DNA and protein synthesis, many other stresses, and to some extent by serum, growth factors, and transforming agents [18]. Two MEK family members, MEK4 (or MKK4) and MEK7 (or MKK7), have been implicated in phosphorylation of JNKs [18]. A number of different MAP3Ks can activate MKK4 and MKK7, suggesting that a wide range of stimuli can activate this MAPK pathway. These include MEKK1, 2, 3, and 4, MLK, and ASK1. In addition to its activation of MKK4 and MKK7, MEKK4 can also activate MKK3 or MKK6 to activate p38 MAPK pathway, which depends on the receptor activated and availability of other signaling molecules [18]. Research into the molecular mechanisms of oxidative stress-mediated activation of JNK and p38 pathways has focused on redox-sensitive proteins such as thioredoxin and glutaredoxin [19]. It is well known that ROS oxidizes thioredoxin to dissociate from ASK-1 for its activation, resulting in the activation of JNK and p38 pathways [20]. 

## 4. MAPK Phosphates

As above mentioned, MAPK pathways are activated through phosphorylation. Thus, the dephosphorylation of MAPKs by phosphatases is likely the most efficient mode of negative regulation. A number of protein phosphatases that are known to deactivate MAPKs include tyrosine, serine/threonine, and dual specificity phosphatases [21, 22]. A group of dual specificity protein phosphatases that are responsible primarily for dephosphorylation/deactivation of MAP kinases are often referred to as MAPK phosphatases (MKPs) [21, 22]. Since MKPs dephosphorylate MAPKs on their regulatory residues, aberrant regulation of MAPK activity may arise through defective regulation of the MKPs. The factors that activate MAPK pathways, such as environmental stresses and growth factor stimulation, can also activate MKP pathways [21, 22], supporting the notion that there is tight and specific control of MAPK activation and function by MKP activation. In mammalian cells, at least 11 MKP family members have been identified so far: MKP-1, MKP-2, MKP-3, MKP-4, MKP-5, MKP-7, MKP-X, PAC1, hVH3, hVH5, and MK-STYX. According to their subcellular localization, MKPs can be grouped: (1) MKP-1, MKP-2, hVH3, and PAC1 are found in the nucleus; (2) MKP-3, MKP-4, and MKP-X are found in the cytoplasm; (3) MKP-5, MKP-7, and hVH5 are found in both the nucleus and the cytoplasm [21, 22]. These MKPs exhibit distinct biochemical properties with regard to their substrate specificity [21, 22]. MKP-1 and MKP-2 show selectivity for p38s and JNKs over ERKs. MKP-3, MKP-X, and hVH3 primarily inactivate ERKs. MKP-5, MKP-7, and hVH5 show selectivity for JNKs and p38s, while MKP-4 and PAC1 inactivate ERKs and p38s. 

MKP-1, the archetype, was initially discovered as a stress-responsive protein phosphatase [23]. Since MKP-1 deactivates MAPKs and is robustly induced by stress stimuli that also activate MAPKs, MKP-1 is regarded as an important feedback control mechanism that regulates the MAPKs. Compared with other MKPs, MKP-1 has been most closely examined. The activity of MKP-1 may be regulated at multiple levels, including transcriptional induction, protein stabilization, catalytic activation, and acetylation [24]. It has been reported that JNK and p38 pathways are highly activated in MKP-1-deficient mouse embryonic fibroblasts [25], supporting that MKP-1 functions as a critical negative regulator during MAPK activation. However, it should be noted that all MPKs may act cooperatively to modulate the MAPK pathways and to orchestrate appropriate cellular responses. 

## 5. Activation of MAPK Pathways by ROS

Studies have demonstrated that ROS can induce or mediate the activation of the MAPK pathways [26]. A number of cellular stimuli that induce ROS production also in parallel can activate MAPK pathways in multiple cell types [4, 26]. The prevention of ROS accumulation by antioxidants blocks MAPK activation after cell stimulation with cellular stimuli [4, 26], indicating the involvement of ROS in activation of MAPK pathways. Moreover, direct exposure of cells to exogenous H_2_O_2_, to mimic oxidative stress, leads to activation of MAPK pathways [27, 28]. The mechanism(s) by which ROS can activate the MAPK pathways, however, is not well defined. Because ROS can alter protein structure and function by modifying critical amino acid residues of proteins [5], the oxidative modification of signaling proteins by ROS may be one of the plausible mechanisms for the activation of MAPK pathways. However, the precise molecular target(s) of ROS is unknown. 

Many growth factor and cytokine receptors have cysteine-rich motifs, the oxidation of which may activate MAPK pathways, and they, if not all, may be targets of oxidative stress. Meng et al. [29] determined the contribution of EGF receptor to activation of ERK pathway by insulin-like growth factor-I (IGF-I) in vascular smooth muscle cells. They showed that IGF-I induced phosphorylation of EGF receptor and ERK. AG1478, an EGF receptor inhibitor, inhibited IGF-I-induced phosphorylation of EGF receptor and ERK, suggesting that activation of ERK pathway results from EGF receptor activation. IGF-I stimulated ROS production and antioxidants inhibited IGF-I-induced ROS generation and activation of EGF receptor and ERK pathway, indicating that IGF-I activates ERK pathway through ROS-mediated activation of EGF receptor. Moreover, Guyton et al. [30] investigated the factors controlling MAPK activation by the oxidant H_2_O_2_. They found that H_2_O_2_ activates MAPK pathways *via* activation of growth factor receptors in several cell types. 

ROS may also activate MAPK pathways through the oxidative modification of intracellular kinases (e.g., MAP3Ks) that are involved in MAPK signaling cascade. ASK-1, a member of the MAP3K superfamily for JNK and p38, binds to reduced thioredoxin in nonstressed cells. Upon an oxidative stress, thioredoxin becomes oxidized and disassociates from ASK-1, leading to activation of JNK and p38 pathways through oligomerization of ASK-1 [31]. A study has been shown that ASK-1-knockout mice exhibited lower levels of JNK and p38 activation in comparison to wild type after oxidant treatment [32]. Besides ASK-1, there may be other redox-sensitive MAP3Ks or MAP2Ks that can also activate MAPK pathways.

Another potential mechanism for MAPK activation by ROS may include the inactivation and degradation of the MKPs that maintain the pathway in an inactive state. Kamata et al. [33] demonstrated that intracellular H_2_O_2_ accumulation inactivates MKPs by oxidation of their catalytic cysteine, which leads to sustained activation of JNK pathway. Hou et al. [34] further confirmed that ROS-induced MKP inactivation causes sustained activation of JNK pathway. Choi et al. [35] showed that glutamate-induced oxidative stress induces sustained activation of ERK pathway through a mechanism that involves degradation of MKP-1. It is worth pointing out that ROS can upregulate MKP-1 expression. Zhou et al. [36] found that upregulation of MKP-1 expression by H_2_O_2_ correlates with inactivation of JNK and p38 activity. Kuwano and Gorospe [37] revealed that the oxidant-triggered induction of MKP-1 is potently influenced by two posttranscriptional processes, mRNA stabilization and increased translation. Lornejad-Schäfer et al. [38] investigated the regulation of MKP-1 expression and JNK activation by the induction of light damage that has shown to enhance ROS production in ARPE-19 cells. In their study, low light doses upregulated MKP-1 expression in ARPE-19 cells, this being accompanied by inactivation of JNK pathway. High light doses, however, led to a decrease in the expression of MKP-1, resulting in sustained activation of JNK pathway. Hence, the paradox in the roles of ROS as “inducers” in the regulation of MKP-1 expression and as “inhibitors” may be, at least in part, related to differences in the concentrations of ROS. 

## 6. Conclusion

**Figure 2:**
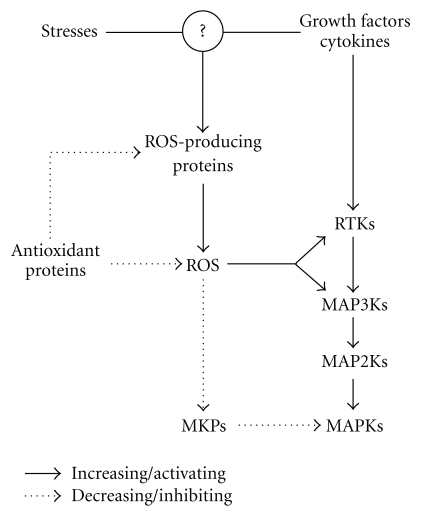
Putative mechanisms for ROS-mediated activation of MAPK pathways. ROS are activated by growth factors, cytokines, and various stresses and rapidly removed by intracellular antioxidant proteins. ROS, once ROS production exceeds the capacity of the antioxidant proteins, may induce oxidative modification of MAPK signaling proteins (e.g., RTKs and MAP3Ks), thereby leading to MAPK activation. ROS may activate MAPK pathways *via* inhibition and/or degradation of MKPs.

The evidence supporting that ROS can activate MAPK pathways at cellular levels is based largely on the following findings: (1) cellular stimuli that are capable of producing ROS can also activate MAPK pathways in a number of different cell types, (2) antioxidants and inhibitors of ROS-producing enzymatic systems block MAPK activation, and (3) exogenous addition of H_2_O_2_, one of ROS, activates MAPK pathways. The putative mechanisms by which ROS, on the basis of their oxidation potentials, can activate MAPK pathways may include (1) oxidative modifications of MAPK signaling proteins (e.g., RTKs and MAP3Ks) and (2) inactivation of MKPs, as illustrated in Figure 2. Finally, the site of ROS production and the concentration and kinetics of ROS production as well as cellular antioxidant pools and redox state are most likely to be important factors in determining the effects of ROS on activation of MAPK pathways.
